# The Impact of Previously Diagnosed Depression on Early and One-Year Mortality in Patients with Acute Ischemic Stroke in Opole Province, Poland

**DOI:** 10.3390/jcm13216576

**Published:** 2024-11-01

**Authors:** Beata Łabuz-Roszak, Anna Starostka-Tatar, Maja Górniak, Kacper Wójcicki, Krzysztof Nalewajko, Robert Zieliński, Mateusz Roszak, Marek Gierlotka

**Affiliations:** 1 Department of Neurology, St. Jadwiga Regional Specialized Hospital, Institute of Medical Sciences, University of Opole, 45-221 Opole, Poland; 2 Department of Nursing, Higher School of Applied Sciences, 41-712 Ruda Slaska, Poland; annastarostka@wp.pl; 3 Student Scientific Association at the Department of Neurology, Institute of Medical Sciences, University of Opole, 45-221 Opole, Poland; gorniak.em@gmail.com (M.G.); mateuszroszakmail@gmail.com (M.R.); 4 Doctoral School, Institute of Medical Sciences, University of Opole, 45-040 Opole, Poland; kawojcicki@gmail.com; 5 Department of Cardiology, University Clinical Hospital, Institute of Medical Sciences, University of Opole, 45-401 Opole, Poland; krzysztof.nalewajko@uni.opole.pl (K.N.); robert.zielinski@uni.opole.pl (R.Z.); marek.gierlotka@uni.opole.pl (M.G.)

**Keywords:** acute ischemic stroke, depression, 30-day mortality, 1-year mortality, post-stroke depression

## Abstract

**Background:** Depression is a known stroke risk factor, but its influence on stroke course depending on gender and age is not clearly defined. The purpose of this study was to determine the impact of previously diagnosed depression on early and one-year mortality in patients with acute ischemic stroke (IS) in relation to gender, age, and concomitant diseases. **Methods:** This study was based on the registry created from the public health insurer in Poland (2009–2020). Two groups were distinguished: IS-D—patients with IS and a diagnosis of depression within preceding 5 years (*n* = 520); IS-nD—patients with IS who had never been diagnosed with depression (*n* = 11,505). **Results:** In-hospital, 30-day, and 1-year mortality did not differ between groups (3.8% vs. 5.1%, 13.1% vs. 12.9%, and 27.1% vs. 26.8%, respectively). However, when statistical analysis was performed stratified by gender and age, we found a significantly higher 30-day and 1-year mortality in men under the age of 65 with previously diagnosed depression in comparison to those without depression (13.6% vs. 3.8%, *p* < 0.001; and 20.3% vs. 10.8, *p* < 0.021, respectively). **Conclusions:** The incidence of post-stroke depression was higher in IS-D group, regardless of gender and age. Early and one-year mortality was higher in younger men with IS and previously diagnosed depression.

## 1. Introduction

According to the World Health Organization (2021), cardiovascular diseases are the leading cause of death worldwide. It is estimated that 32% of all deaths are caused by cardiovascular diseases [1]. Among them is stroke, the main cause of permanent disability in adults in Europe. Due to its poor prognosis, and the high costs of treatment and of chronic care, stroke is not only a medical but also a social problem. There are numerous analyses of risk factors for cardiovascular diseases, based on which guidelines for the treatment and prevention of stroke are formulated, which led to a 12% reduction in mortality in the years 2010–2020 [2]. Of the mental diseases analyzed in terms of their potential impact on the occurrence of cardiovascular diseases, depression is especially pertinent.

The impact of depression on cardiovascular diseases is being studied by many centers around the world. Considering the increase in the prevalence of depression by almost 50% in the last 30 years, such studies are extremely necessary [3]. According to data provided by the World Health Organization, about 5% of adults suffer from depression, and in the population over 60 years of age it is 5.7%. It is estimated that around 280 million people worldwide suffer from depression [4]. Untreated depression has health and behavioral consequences, affects personal, professional, and social life, and thus the quality of life and well-being of the patient. It also affects the quality of life of family members and friends of the person suffering from depression. This is a very important health and social problem of the 21st century which requires prevention, detection, and management [5].

Depression is a known cardiovascular risk factor. Chinese authors indicate a 30% increase in the risk of cardiovascular diseases in patients with depression [6], while Italian authors, based on international reports, describe a risk increased by as much as 72% [7]. An American analysis with data from almost two million patients, has shown that depression increases the risk of myocardial infarction by 1.28 times and stroke by 1.13 times [8]. In the INTERHEART study, the four most important factors associated with acute coronary syndromes were dyslipidemia, smoking, psychosocial factors (predominantly depression, stress, life events, and locus of control), and diabetes [9,10]. In the INTERSTROKE study, depression is listed as one of ten potentially modifiable factors which are associated with a 90% risk of stroke [11,12].

There are many discussed potential biological mechanisms that link depression with cardiovascular diseases, such as disturbances of the autonomic nervous system, platelet receptors and function, coagulopathic factors, proinflammatory cytokines, endothelial function, neurohormonal, and genetic factors [9,13,14,15,16]. Moreover, depression is associated with poor adherence to medical treatment (including taking cardiovascular drugs) [9,17].

While it is indisputable that depression is a risk factor for stroke, the impact of preexisting mood disorders on the course of stroke is not clearly defined. Although there are some reports on association between preadmission depressive symptoms and poorer functional outcome [18], the relationship between a history of depression and mortality after acute stroke depending on gender and age is inconclusive.

Therefore, the purpose of the study was to determine the impact of previously diagnosed depression on early and long-term mortality in patients with acute ischemic stroke (IS) in relation to gender, age, and concomitant diseases.

## 2. Materials and Methods

The study was based on the Opole Database of Civilization Diseases registry created from the administrative database of the only public and obligatory health insurer in Poland (National Health Fund, NHF). The registry database is operated by the Department of Cardiology of University Hospital in Opole, based on an agreement with the Regional Department of NHF in Opole, Poland. The registry included all information regarding health services for a particular patient in the database. All data were anonymized. However, matching information concerning the individual patient was possible through unique numbers in the database.

The data from neurological and stroke departments in Opole Province were taken into account as well as those from all medical services reported to the NHF, both hospital and outpatient, in years 2009–2020. Opole Province is the smallest region located in southern Poland, inhabited by 942,441 people (as of 31 December 2022, according to the Central Statistical Office), representative for the entire country.

The IS cases were selected from the whole registry based on primary diagnosis coded as I63 in the International Classification of Diseases version 10 (ICD-10) (*n* = 12,025). Next, among these, cases with a diagnosis of depression both in in-patients and out-patients services (coded as F32 and/or F33 in ICD-10) within 5 years before stroke onset (*n* = 520) were distinguished. Moreover, the following variables from the registry were considered: age, gender, concomitant diseases diagnosed before stroke onset (hypertension, diabetes mellitus, previous ischemic stroke, coronary artery disease, atrial fibrillation, heart failure, cancer), hospitalization duration, in-hospital mortality, 30-day mortality, and 1-year mortality. The concomitant diseases were based on diagnoses reported to the NHF in Opole from hospitalization as well as from ambulatory care.

One-year follow-up was carried out to search for the diagnosis of post-stroke depression.

Ethical review and approval were waived for this study because it was based on retrospective anonymous data taken from registry created from the administrative database. This study was not a medical experiment.

### Statistical Analysis

Data were presented as either mean + standard deviation, median and interquartile range (IQR), or count and percentages. The type of distribution was verified using the Shapiro–Wilk test. Differences between groups were compared using Student’s *t*-test for normally distributed variables, the Mann–Whitney U-test for non-normally distributed variables, and the χ^2^ test for the nominal variables. A two-sided p-value of less than 0.05 was considered significant. All analyses were conducted using the R software v. 4.2.2 (The R Foundation for Statistical Computing, Vienna, Austria).

## 3. Results

From the registry, 12,025 cases with IS (I63) were analyzed. Two groups were created:

(1) IS-D: patients with IS and a diagnosis of depression within preceding 5 years (*n* = 520), and
(2) IS-nD: patients with IS who had never been diagnosed with depression according to the available data in the registry (*n* = 11,505).

The mean age in both groups was similar (72.4 ± 11.6 vs. 72.3 ± 12.3 years), while the percentage of women was higher in the IS-D group (71% vs. 50%; *p* < 0.001) (Table 1).

Hospitalization duration did not differ between groups (3.8 ± 8 days vs. 4.9 ± 22 days; NS). Hypertension (*p* < 0.001), cancer (*p* = 0.006), diabetes mellitus (*p* = 0.007), and coronary artery disease (*p* < 0.001) were more common in the IS-D group. We found no difference in the incidence of atrial fibrillation, heart failure, and previous myocardial infarction between groups (Table 2).

In-hospital, 30-day, and 1-year mortality did not differ significantly between groups (3.8% vs. 5.1%, 13.1% vs. 12.9%, and 27.1% vs. 26.8%, respectively). However, when statistical analysis was performed stratified by gender and age, we found a significantly higher 30-day and 1-year mortality in younger men (<65 years old) with IS and prior depression in comparison to those without depression (13.6% vs. 3.8%, *p* < 0.001; and 20.3% vs. 10.8, *p* < 0.021, respectively) (Table 3 and Table 4).

The incidence of post-stroke depression (PSD) was higher in the IS-D group (*n* = 75; 14.4%) in comparison to IS-nD group (*n* = 68; 0.6%) (*p* < 0.001), regardless of gender and age.

## 4. Discussion

Our study reveals that early and one-year mortality was higher in younger men (under the age of 65 years) with acute ischemic stroke and previously diagnosed depression. However, we found no effect of previously diagnosed depression on mortality rates in women or older men with stroke. The results of other authors regarding the relationship between a history of depression and prognosis after stroke and the dependence on gender and age are ambiguous. Meng et al. observed a higher mortality rate due to cardiovascular events in patients with previously diagnosed depression [19]. Chinese studies, covering a population of about 500,000 of patients, showed higher mortality in men in the course of cardiovascular diseases with accompanying depression, which is consistent with the data obtained in our study [20]. Another Chinese study indicates even a 55% higher mortality in patients with depression in the course of stroke [21]. The phenomenon of increased risk of cardiovascular diseases or mortality in the course of these diseases in patients diagnosed with depression may be the result of various biochemical processes. In a Japanese study, about one million people with type 2 diabetes and depression were assessed and an increased risk of cardiovascular diseases was shown, although the results may be controversial, because diabetes itself increases this risk [22]. A similar study from an Italian center focuses on biomarkers found in people with insulin resistance—specifically PCSK9—and the potential effect of depression on the increase in this marker. Such an effect is possible, but the unequivocal effect of depression on its increase has not been proven [23]. Depression is believed to increase the risk of cardiovascular diseases by activating the immune system, increasing the value of inflammatory parameters and, consequently, the endothelial dysfunction and activation of the coagulation system. In addition, depression is a cause of stress, which activates the sympathetic nervous system, which increases the heart rate and may lead to cardiac hypertrophy and, as a consequence, vascular diseases [24]. Imbalance in the NLRP3 (NOD-like receptor family, pyrin domain containing 3) inflammasome in critical brain areas involved in pain perception, like the prefrontal cortex and hippocampus, may contribute to the development of mood disorders following spinal cord injury [25]. Depression also modifies the hypothalamic–pituitary–adrenal axis, which increases the production of cortisol, which may lead to metabolic syndrome, which is also a risk factor for cardiovascular diseases [26].

Scientists are also interested in the different mortality rates of patients with depression in the course of cardiovascular diseases depending on gender. So far, data on the cause of these differences are not certain, but it is believed that this phenomenon may be influenced by differences in the content of antioxidants in mitochondria, which is higher in women, thanks to which the phenomenon of oxidative stress causes less cell destruction in women [19]. Another reason considered is the earlier treatment of depression in women, who, compared to men, more often report to a psychiatrist in the early stages of the disease [27]. Other possible causes have also been described, such as differences in the biochemical mechanisms of the central nervous system in women and men, but this issue requires further research [28]. Studies are also being conducted to assess the cardiovascular benefits of treating depression. So far, data on the effect of pharmacological treatment of depression on reducing the risk of cardiovascular diseases vary depending on the center. Chinese data do not describe such an effect, although this may be due to the low percentage of people treated for depression in China [29], while Australian authors show a reduction in risk in men effectively treated pharmacologically for depression; however, no reduction in cardiovascular risk has been demonstrated in people treated with antidepressants for other reasons [30]. American researchers also address this issue, arguing that people with effective depression treatment live longer, but without demonstrating a direct effect of antidepressant treatment on reducing the risk of cardiovascular disease. Despite this, depression treatment has a beneficial effect on other aspects of therapy for patients, who are more enthusiastic about the treatment of other diseases and are more willing, for example, to change their lifestyle to a more active one or to change their diet [31,32]. British data on the effect of psychotherapy on the risk of cardiovascular disease present a slightly different perspective. They show that in patients who improved after psychotherapy, the risk of cardiovascular incidents in the next three years is about 12% lower than in patients whose mental state was not improved by psychotherapy. This applies especially to patients under 60 years of age [33]. Also interesting are the observations of researchers from the University of Cambridge, who assessed the risk of cardiovascular disease in correlation with depression in patients up to 24 years of age. In this age group, the relationship is unclear, but depression may be associated with increased BMI, smoking, and poorer glycemia control in patients with diabetes. All these circumstances are potential risk factors for cardiovascular diseases [34]. Regardless of the cause, cardiovascular disease prevention, consisting of physical activity, is beneficial. It also has a positive effect on mood [35]. All these data show how important a holistic approach to the patient is, which is not limited to their physical condition but also includes their emotional state.

In summary, the novelty of our study was to find an association between 30-day and 1-year mortality in men under 65 years of age after acute ischemic stroke who were previously diagnosed with depression. We did not find this association in women, regardless of age, or in older men (over 65 years of age). Our observations require further studies to clearly establish the relationship between depression and prognosis after acute stroke.

## 5. Limitations

There were a few limitations to this research, as described in our previous studies [36,37,38,39]. Firstly, it is an analysis of administrative, health insurer database associated with reimbursement, not a prospective clinical registry. Secondly, the diagnosis of IS, according to ICD-10 codes, was not additionally verified and thus, human mistakes or improper diagnoses are possible. Thirdly, no additional clinical data were available for the analyses, such as clinical history, the etiology and severity of the stroke, functional status, or pharmacological treatment. Fourthly, during follow-up observation, some patients may have permanently migrated outside the Opole Province and been lost to follow-up; however, this effect should be marginal in the population after stroke. This limitation does not apply to mortality, which was available for all cases, independently of the place of residence. Fifthly, ambulatory care could have been partially provided by private visits not reported to the health insurance system.

## 6. Conclusions

Early and one-year mortality was higher in men under the age of 65 years with acute ischemic stroke and previously diagnosed depression. Therefore, in patients with IS a special attention should be paid to the presence of depression in medical history, as it can affect prognosis, especially in younger men. Moreover, the previous depression in patients with ischemic stroke increases the risk of post-stroke depression.

## Figures and Tables

**Table 1:** Characteristics of the examined groups.

	IS-nD Group	IS-D	*p*
Number of patients	11,505	520	
Female, *n* (%)	5756 (50%)	370 (71.2%)	<0.001
Male, *n* (%)	5749 (50%)	150 (28.8%)	
Age, X ± SD, years	72.3 ± 12	72.4 ± 11	0.880

IS-nD: patients with IS had never been diagnosed with depression according to the available data in the registry; IS-D: patients with IS and a diagnosis of depression within the preceding 5 years.

**Table 2:** Prevalence of concomitant diseases in examined groups.

Disease	IS-D Group (N = 520) *n* (%)	IS-nD Group (N = 11,505) *n* (%)	*p*
Hypertension	465 (89.4%)	9606 (83.5%)	<0.001
Atrial fibrillation	117 (22.5%)	2271 (19.7%)	0.123
Previous myocardial infarction	38 (7.3%)	979 (8.5%)	0.335
Coronary artery disease (without myocardial infarction)	253 (48.7%)	4543 (39.5%)	<0.001
Diabetes mellitus	213 (41.0%)	4049 (35.2%)	0.007
Cancer	85 (16.3%)	1416 (12.3%)	0.006
Heart failure	159 (30.6%)	3513 (30.5%)	0.984

IS-nD: patients with IS who had never been diagnosed with depression according to the available data in the registry; IS-D: patients with IS and a diagnosis of depression within the preceding 5 years.

**Table 3:** Mortality in men (IS-D vs. IS-nD).

**Men ≤ 65 Years Old**			
	**IS-D (N = 59)** ***n* (%)**	**IS-nD (N = 1891)** ***n* (%)**	***p***
In-hospital mortality	1 (1.7%)	25 (1.3%)	0.809
30-day mortality	8 (13.6%)	72 (3.8%)	<0.001
1-year mortality	12 (20.3%)	204 (10.8%)	0.021
**Men > 65 and <75 years old**			
	**IS-D (N = 42)** ***n* (%)**	**IS-nD (N = 1860)** ***n* (%)**	***p***
In-hospital mortality	1 (2.4%)	80 (4.3%)	0.542
30-day mortality	3 (7.1%)	177 (9.5%)	0.603
1-year mortality	7 (16.7%)	372 (20.0%)	0.593
**Men ≥ 75 years old**			
	**IS-D (N = 49)** ***n* (%)**	**IS-nD (N = 1998)** ***n* (%)**	***p***
In-hospital mortality	2 (4.1%)	131 (6.6%)	0.487
30-day mortality	9 (18.4%)	349 (17.5%)	0.870
1-year mortality	19 (38.8%)	735 (36.8%)	0.776

IS-nD: patients with IS who had never been diagnosed with depression according to the available data in the registry; IS-D: patients with IS and a diagnosis of depression within the preceding 5 years.

**Table 4:** Mortality in women (IS-D vs. IS-nD).

**Men ≤ 65 years old**			
	**IS-D (N = 76)** ***n* (%)**	**IS-nD (N = 981)** ***n* (%)**	***p***
In-hospital mortality	1 (1.3%)	23 (2.3%)	0.562
30-day mortality	3 (3.9%)	48 (4.9%)	0.711
1-year mortality	10 (13.2%)	103 (10.5%)	0.470
**Men > 65 and <75 years old**			
	**IS-D (N = 85)**	**IS-nD (N = 1210)** ***n* (%)**	***p***
In-hospital mortality	3 (3.5%)	37 (3.1%)	0.808
30-day mortality	9 (10.6%)	93 (7.7%)	0.337
1-year mortality	12 (14.1%)	204 (16.9%)	0.512
**Men ≥ 75 years old**			
	**IS-D (N = 209)** ***n* (%)**	**IS-nD (N = 3565)** ***n* (%)**	***p***
In-hospital mortality	12 (5.7%)	294 (8.2%)	0.197
30-day mortality	36 (17.2%)	744 (20.9%)	0.206
1-year mortality	81 (38.8%)	1460 (41.0%)	0.530

IS-nD: patients with IS who had never been diagnosed with depression according to the available data in the registry; IS-D: patients with IS and a diagnosis of depression within the preceding 5 years.

## Data Availability

The data presented in this study are available on request from the corresponding author. The data are not publicly available due to privacy reasons.
